# Enhancing Constitutive Description of 5A06 Aluminum Alloy During Warm Deformation Using Machine Learning-Assisted Johnson–Cook Model

**DOI:** 10.3390/ma19142987

**Published:** 2026-07-10

**Authors:** Zhao Liu, Lei Deng, Jinchuan Long, Chang Gao, Yi Hao, Pan Gong, Xuefeng Tang, Xinyun Wang

**Affiliations:** 1 State Key Laboratory of Materials Processing and Die & Mould Technology, Huazhong University of Science and Technology, No. 1037, Luoyu Road, Hongshan District, Wuhan 430074, China; d202080413@hust.edu.cn (Z.L.); d202080373@hust.edu.cn (C.G.); d202080350@hust.edu.cn (Y.H.); pangong@hust.edu.cn (P.G.); wangxy_hust@hust.edu.cn (X.W.); 2 School of Intelligent Manufacturing and Robotics, Shanghai DianJi University, No. 300, Shuihua Road, Pudong New District, Shanghai 201306, China; 3 State Key Laboratory of Precision Manufacturing for Extreme Service Performance, School of Mechanical and Electrical Engineering, Central South University, Changsha 410083, China; longjc1226@csu.edu.cn

**Keywords:** 5A06 aluminum alloy, warm deformation behavior, artificial neural network, constitutive modeling, thermal softening

## Abstract

**Highlights:**

- Novel MOI-ANN-JC model accurately predicts flow stress of 5A06 aluminum alloy during warm deformation.
- Dynamic tracking of thermal softening exponent effectively overcomes the limitations of traditional processing maps.
- Embedded FEA successfully predicts localized softening and macroscopic fracture defects in actual forging components.

**Abstract:**

To accurately characterize the warm deformation behavior and workability of the 5A06 aluminum alloy, this study presents an innovative workflow that develops and systematically validates machine learning-assisted Johnson–Cook (ML-JC) frameworks based on artificial neural network (ANN) surrogate models. Two predictive frameworks—the parallel-decoupled PD-ANN-JC and the multi-objective integrated MOI-ANN-JC—were constructed. Quantitatively, both developed ML-JC frameworks achieve significantly higher stress prediction accuracy and superior generalization capability compared with the conventional JC model. Specifically, on the testing set, the MOI-ANN-JC framework yields an average absolute relative error (AARE) of 1.424% and an *R*^2^ of 0.997, outperforming the PD-ANN-JC framework (AARE of 3.246%, *R*^2^ of 0.988). On the validation set, the MOI-ANN-JC framework also demonstrates exceptional generalization, with an AARE of 3.302% and an *R*^2^ of 0.987. Scientifically, the superior performance of the MOI-ANN-JC framework stems from its ANN-*mnδ* surrogate model, which simultaneously predicts the strain hardening exponent *n*, thermal softening exponent *m*, and relative error *δ* directly from deformation parameters. This mutual coupling establishes an intrinsic correlation between *m* and *n*, successfully aligning with the physical reality wherein strain hardening and thermal softening are inherently linked during deformation. Qualitatively and practically, by integrating the MOI-ANN-JC framework into finite element (FE) simulation software, dynamic tracking and visualization of the thermal softening exponent *m* during warm deformation were achieved. Combined with FE simulations, Vickers hardness testing and EBSD observations, this study successfully establishes a direct qualitative spatial correspondence between low-*m* regions and macroscopic defects, which was further verified through the warm forging of a thin-walled dual-cavity component. Crucially, this approach for evaluating deformation stability bridges the gap caused by the inapplicability of conventional processing maps within this temperature regime, offering a robust and broadly applicable workflow for complex forming optimization.

## 1. Introduction

5A06 aluminum alloy belongs to the Al-Mg (5xxx series) system and is extensively employed across various industrial sectors owing to its low density, favorable hardness, and excellent weldability [1,2,3]. Because of the sluggish diffusion rate of magnesium atoms within the aluminum matrix, these alloys are typically classified as non-heat-treatable, relying primarily on work hardening as their predominant strengthening mechanism [4,5]. Performing deformation in the warm working regime can effectively suppress dislocation recovery [6,7], enabling the strength to approach that of the T6 temper [8]. Moreover, this processing strategy alleviates localized thinning [9] and mitigates surface damage [10], thereby significantly enhancing overall formability. An example was reported by Liu et al. [11], who demonstrated that the warm deformation of complex thin-walled 5A06 components yielded remarkable improvements in both structural integrity and dimensional precision. To facilitate the widespread industrial adoption of this technology, an accurate description of the complex flow behavior must be intrinsically coupled with reliable predictions of material workability.

To accurately characterize the flow behavior, the temperature regime for warm deformation in aluminum alloys must first be precisely demarcated. A conventional macroscopic approach categorizes the processing regime based on the homologous temperature (*T_H_* = *T*/*T_m_*, where *T_m_* is the absolute melting point) [12]. Under this criterion, warm deformation is typically identified by a *T*_H_ ranging from 0.3 to 0.5, corresponding to an actual deformation temperature between 0.3*T_m_* and 0.5*T_m_*, wherein the flow stress is governed by the dynamic competition between work hardening and thermal softening, exhibiting characteristic elastic–viscoplastic behavior. Once the temperature exceeds 0.5*T_m_*, the material progressively transitions into a fully viscoplastic hot working state, where thermal softening mechanisms assume predominant control over the flow behavior [13]. Alternatively, a microstructural approach delineates the upper boundary of warm deformation strictly below the recrystallization temperature [14]. Consolidating these two criteria, the present study defines the warm deformation of aluminum alloys as the regime exceeding 0.3*T_m_* yet remaining below the recrystallization isotherm (approximately from room temperature to 300 °C).

Characterizing such complex mechanical responses requires appropriate constitutive modeling. Given the non-fully viscoplastic nature of aluminum alloys within this warm regime, traditional high-temperature creep models (such as Arrhenius-type equations) that are predicated on viscoplasticity and inherently neglect strain hardening are fundamentally inadequate [15,16,17,18]. In contrast, the Johnson–Cook (JC) model does not rely on the fully viscoplastic assumption; instead, it incorporates strain, strain rate, and temperature dependencies, comprehensively accounting for the combined effects of strain hardening and thermal softening [19,20]. Furthermore, because the JC framework adopts the lowest experimental temperature as its lower baseline [21], its applicable temperature span is effectively broadened [22,23], making it well-suited for describing the warm deformation behavior.

Nevertheless, the standard JC model relies on static empirical constants, which fail to capture the dynamically fluctuating material properties caused by complex microstructural evolution during deformation, ultimately leading to a noticeable degradation in predictive precision. Researchers have attempted to resolve this by replacing static constants with sophisticated strain- or temperature-dependent functions [19]. For instance, Lin et al. [24] optimized the constitutive framework by incorporating the coupled effects of temperature and strain rate on flow behavior while preserving the original yield stress and strain hardening terms. Similarly, Zhang et al. [25] introduced polynomial expressions to account for variable thermal and hardening rates. Although these mathematical modifications enhance flow stress prediction accuracy, they often degrade the constitutive equation into a purely empirical fitting tool, sacrificing the explicit physical significance of the original parameters [26,27]. While dynamically updating the physically meaningful parameters of the classical JC model across varying deformation conditions represents a much more desirable strategy, the evolution of these parameters is synergistically governed by highly non-linear, strongly coupled thermal and mechanical effects. Traditional optimization algorithms based on simplified analytical functions or multivariate linear regressions are incapable of navigating such complex, high-dimensional parameter spaces with high precision.

Artificial neural networks (ANNs) offer a powerful alternative, as they bypass complex analytical derivations to directly extract deep nonlinear mapping relationships from massive datasets [28,29,30]. In fact, ANNs have even demonstrated the capability to directly predict flow stress based solely on deformation parameters; Sudhy et al. [31] trained ANN models for AA2014, AA5052, and AA6082 alloys, achieving correlation coefficients exceeding 0.99 between experimental measurements and model predictions across all cases. However, this pure “black-box” approach completely obscures the underlying physical mechanisms. To bridge this gap, Long et al. [32] coupled an ANN with a reformulated Arrhenius model containing a relative error term *δ* to characterize the hot forming of magnesium alloys. Their network mapped deformation conditions directly to both the thermal activation energy (*Q*_act_) and *δ*, which were subsequently fed back into the constitutive equation to predict flow stress. By dynamically tracking the parametric dependency of *Q*_act_ to reflect the underlying microstructural evolution, such a hybrid framework successfully demonstrated that precise parameter optimization and physical interpretability can be harmoniously unified. Despite its success in hot working regimes, analogous research focusing on the warm deformation of aluminum alloys remains scarcely reported.

Beyond flow stress modeling, evaluating material workability represents the other critical pillar of forming optimization. For hot deformation, constructing processing maps to calculate power dissipation efficiency (*η*) and predict flow instability domains is a widely adopted methodology. While investigators such as Gao et al. [33] and Wen et al. [28] have successfully utilized hot processing maps to predict dynamic recrystallization, optimize thermomechanical parameters, and guide microstructural tailoring in alloys such as 2024 aluminum and powder metallurgy superalloys, inherent limitations persist. Practically, the safe processing domains identified by conventional maps frequently overestimate the actual viable manufacturing windows [34]; for example, when applied to guide parameter optimization for 5052 aluminum alloy, traditional processing maps fail to accurately capture the formation of detrimental chain-like microstructures [35]. More fundamentally, from a theoretical perspective, constructing processing maps is fundamentally based on the Dynamic Materials Model (DMM), which is predicated on the assumption of a fully viscoplastic [36]. Consequently, within the temperature regime investigated in this work, the aluminum alloy does not satisfy the fully viscoplastic assumption, rendering traditional processing maps inapplicable, highlighting a need for alternative predictive methodologies.

To overcome these dual challenges in flow characterization and defect prediction, this study developed a Machine Learning-Assisted Johnson-Cook (ML-JC) model for 5A06 aluminum alloy during warm deformation, achieving both precise flow stress prediction and the dynamic calibration of physically meaningful parameters. Building upon this hybrid framework, and integrating finite element simulations with physical forging trials, a novel methodology for predicting warm deformation defects in Al-Mg alloys by dynamically tracking the evolution of the thermal softening index was proposed.

## 2. Materials and Methods

The 5A06 aluminum alloy investigated in this study was provided by Dongqing Aluminum Industry Co., Ltd. (Harbin, China) in the form of a rolled thick plate. Prior to testing, artificial aging treatment was conducted to stabilize the initial microstructure. The chemical composition of the alloy is summarized in Table 1.

**Figure 1:**
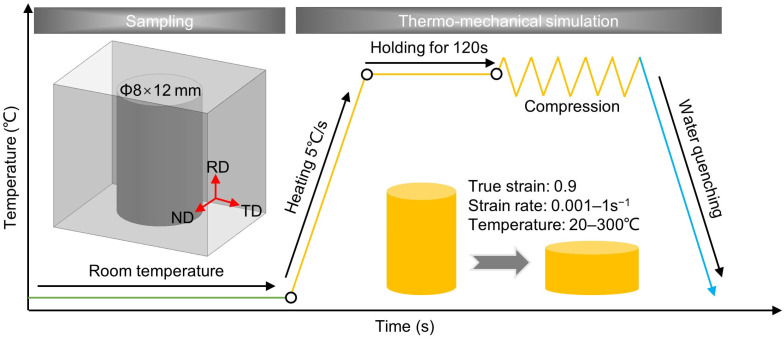
Schematic of the isothermal compression test: specimen sampling and experimental procedures.

To investigate the warm deformation behavior of the 5A06 aluminum alloy, an isothermal compression testing scheme was implemented, as illustrated in Figure 1. Cylindrical specimens with dimensions of Φ8 mm × 12 mm were machined from the as-received thick plate, with their longitudinal axes aligned parallel to the rolling direction (RD). Compression tests were conducted using a Gleeble-3500 thermal-mechanical simulator (DSI, Poestenkill, NY, USA) across a temperature range of 20–300 °C (with increments of 50 °C) and strain rates of 0.001, 0.01, 0.1, and 1 s^−1^. The specimens were compressed to a total height reduction of 60%, corresponding to a maximum true strain of approximately 0.9. Following deformation, all specimens were immediately water-quenched to preserve the as-deformed microstructure for subsequent analysis.

For the Vickers hardness tests, specimens were mechanically polished, followed by measurements using a Vickers hardness tester (Beijing TIME High Technology Ltd., Beijing, China) with a load of 200 gf and a dwell time of 10 s. An electron backscattered diffraction (EBSD) detector equipped on a field-emission scanning electron microscope (FE-SEM) (JEOL, Tokyo, Japan) was utilized to analyze the microstructural evolution. EBSD specimens were prepared via mechanical polishing followed by electropolishing in a solution of 10 vol.% perchloric acid and 90 vol.% anhydrous ethanol at 20 V for 20 s.

## 3. Results and Discussion

### 3.1. Constitutive Model for the Warm Deformation of 5A06 Aluminum Alloy

#### 3.1.1. Conventional JC Model

**Figure 2:**
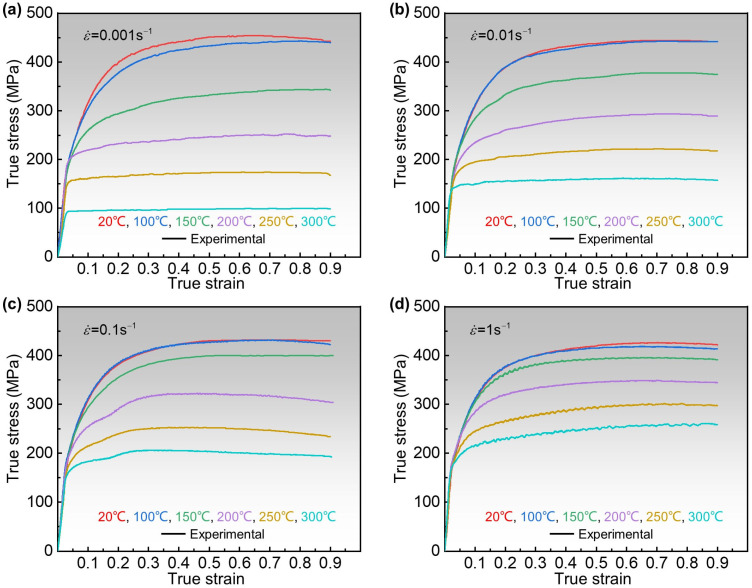
True stress-strain curves of 5A06 aluminum alloy at temperatures ranging from 20 to 300 °C under strain rates of: (**a**) 0.001 s^−1^, (**b**) 0.01 s^−1^, (**c**) 0.1 s^−1^, and (**d**) 1 s^−1^.

Figure 2 presents the true stress-strain curves of the 5A06 aluminum alloy obtained from isothermal compression tests. Across all conditions, the flow stress initially rises sharply with increasing strain due to work hardening and subsequently maintains a steady-state plateau, showing no pronounced macroscopic softening typically observed in hot deformation [32].

The conventional Johnson–Cook (JC) [37] model is phenomenologically expressed as the product of three independent terms, representing the effects of strain hardening, strain rate strengthening, and thermal softening, respectively. The model is formulated as follows:

(1) σ=A+Bεn1+Clnε˙ε˙r1−T−TrTm−Trm

where *ε* is the true strain, $\dot{\varepsilon }$ is the strain rate, and ${\dot{\varepsilon }}_{r}$ represents the reference strain rate. *T* is the deformation temperature, *T_r_* and *T_m_* denote the reference temperature and the melting point of the material, respectively. Unlike the homologous temperature expression that necessitates using the absolute melting point for *T_m_*, the JC model allows *T_m_* to be expressed in either Kelvin or Celsius, provided that its units are uniform with those of *T* and *T_r_*. The material constants to be identified include *A* (initial yield stress at *T_r_* and ${\dot{\varepsilon }}_{r}$), *B* (strain hardening modulus), *C* (strain rate sensitivity coefficient), *n* (hardening exponent), and *m* (thermal softening exponent) [16,38]. Prior to parameter identification, it is essential to establish the reference conditions, which are typically defined by the lowest experimental temperature and strain rate [39]. Accordingly, 20 °C and 0.001 s^−1^ were selected as the reference temperature and strain rate in this study.

**Figure 3:**
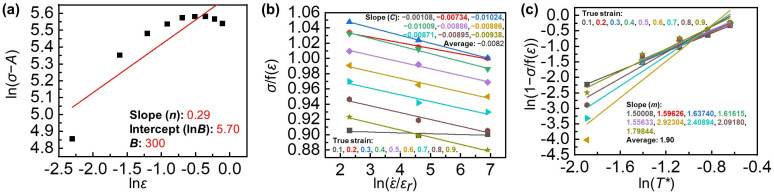
Fitting process of parameters (**a**) *n* and *B*, (**b**) *C*, and (**c**) *m* in the conventional JC model.

The constitutive model established in this study is primarily intended to characterize the flow behavior of the material during bulk forming processes. Given that the accumulated true strain in practical applications, such as forging and extrusion, generally exceeds 0.1, experimental data within the true strain range of 0.1 to 0.9 were selected for parameter identification. The identification procedure for the conventional JC model parameters is schematically illustrated in Figure 3, Where T* = (*T* − *T_r_*)/(*T_m_* − *T_r_*) The resulting JC constitutive model, incorporating the fitted constants, is presented in Equation (2), with the specific numerical values of the parameters summarized in Table 2.

(2) σJC=188+300ε0.291−0.0082lnε˙ε˙r1−T−TrTm−Tr1.9

**Figure 4:**
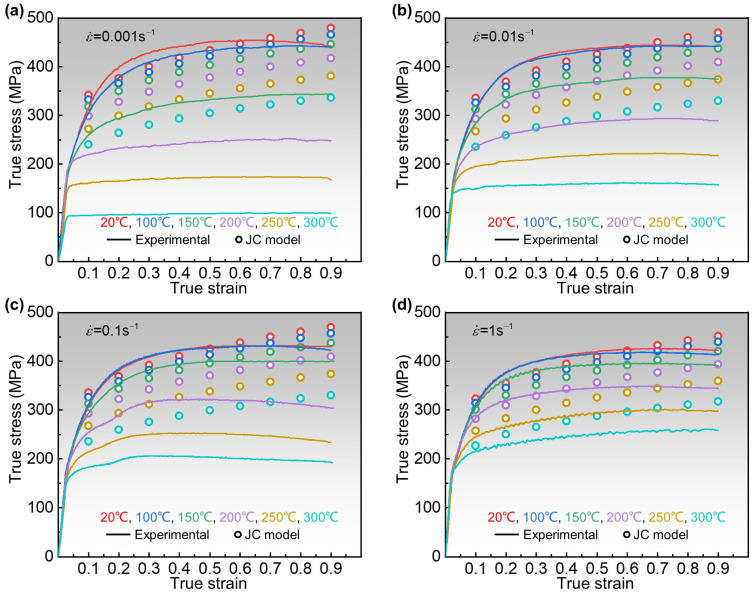
Comparison between the predicted flow stress from the conventional JC model and the experimental results under strain rates of: (**a**) 0.001 s^−1^, (**b**) 0.01 s^−1^, (**c**) 0.1 s^−1^, and (**d**) 1 s^−1^.

To evaluate the predictive capability of the established conventional JC model, flow stress values were calculated using Equation (2), encompassing true strains of 0.1–0.9, strain rates of 0.001–1 s^−1^, and temperatures of 20–300 °C. As illustrated in Figure 4, throughout the entire deformation process, significant discrepancies exist between the predictions of the conventional JC model and the experimental curves. Specifically, within the lower strain range (about 0.1 to 0.5), the flow stress predicted by the JC model increases with increasing strain, which is consistent with the experimental trend. However, in the higher strain range (about 0.5 to 0.9), the experimental curves gradually flatten out or even exhibit a downward trend as strain increases. This phenomenon indicates a competition between work hardening and softening mechanisms within the material. In contrast, the conventional JC model predicts a continuous increase in flow stress, failing to capture this hardening-softening competition. This is consistent with previous reports indicating that the constant parameters in the constitutive model fail to represent the continuously changing material properties during deformation [40].

**Figure 5:**
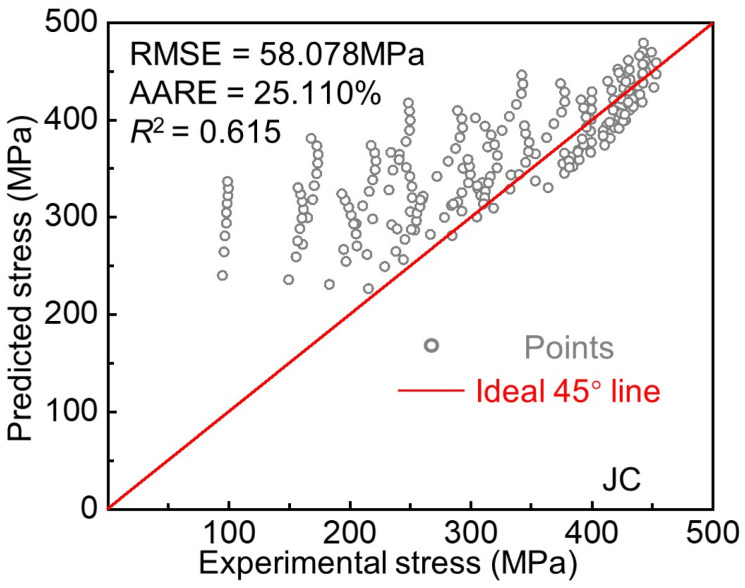
Correlation between experimental and predicted results using the conventional JC model.

To quantitatively evaluate the predictive performance of the models mentioned in this study, several statistical metrics were employed, including the coefficient of determination (*R*^2^, Equation (3)), the root mean square error (RMSE, Equation (4)), the average absolute relative error (AARE, Equation (5)), and the relative error (*δ*, Equation (6)). These metrics were calculated as follows:

(3) $$R^{2}=1-\frac{\sum_{i=1}^{N}{\left(P_{i}-E_{i}\right)}^{2}}{\sum_{i=1}^{N}{\left(\bar{E}-E_{i}\right)}^{2}}$$

(4) $$\mathrm{RMSE}=\sqrt{\frac{1}{N}\sum_{i=1}^{N}{\left(P_{i}-E_{i}\right)}^{2}}$$

(5) $$\mathrm{AARE}=\frac{100\%}{N}\sum_{i=1}^{N}\left|\frac{P_{i}-E_{i}}{E_{i}}\right|$$

(6) $$\delta =\frac{E_{i}-P_{i}}{E_{i}}\times 100\%$$

where *E_i_* and *P_i_* represent the experimental value and the predicted value at point *i*, respectively. $\bar{E}$ represents the arithmetic mean of all experimental values *E_i_*. Figure 5 presents the linear fitting results between the values predicted by the conventional JC model and the experimental data. The calculated RMSE, AARE, and *R*^2^ are 58.078 MPa, 25.110%, and 0.615, respectively.

The maximum absolute relative errors (ARE) (|*δ*|_max_) under various strain rates were calculated from the data in Figure 4 and are summarized in Table 3. The peak relative error reaches 70.62%. Both visual curve comparisons and quantitative statistical metrics confirm that the conventional JC model yields poor predictive accuracy when characterizing the warm deformation flow stress of the 5A06 aluminum alloy.

#### 3.1.2. Machine Learning-Assisted JC Model

**Figure 6:**
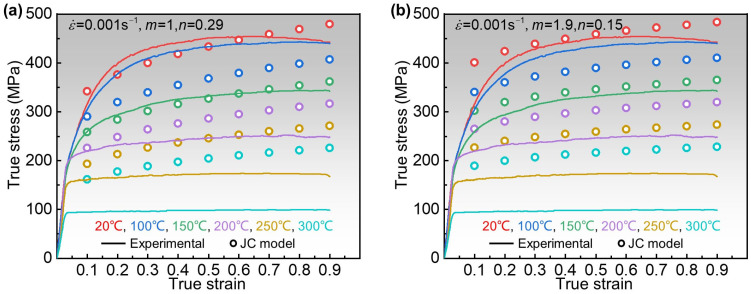
Comparisons between the experimental results and the model predictions following the modification of: (**a**) the thermal softening exponent *m* and (**b**) the hardening exponent *n*.

Given that the experimental curves exhibit a dynamic competition between work hardening and softening mechanisms, it is postulated that optimizing the thermal softening exponent (*m*) and the hardening exponent (*n*) in the conventional JC model could improve the agreement between the predicted and experimental curves. To systematically investigate the sensitivity of model predictions to *m* and *n*, a comparative analysis was performed based on the initial values listed in Table 2. Specifically, the value of *m* was adjusted to 1.0 while keeping *n* constant; similarly, *n* was reduced to 0.15 with *m* remaining unchanged. The flow stress was then recalculated at a strain rate of 0.001 s^−1^. The resulting comparisons between the modified predictions and the experimental data are illustrated in Figure 6a and Figure 6b, respectively.

**Figure 7:**
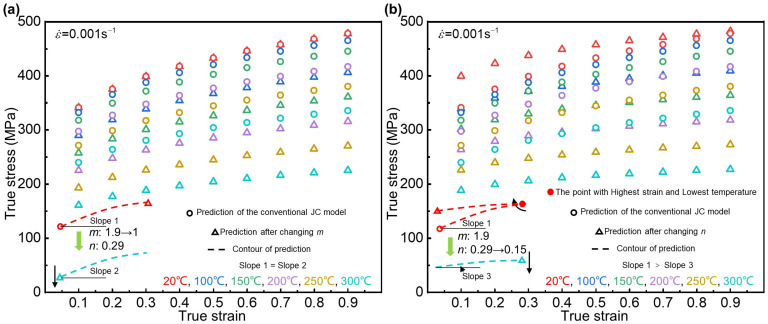
Effects of individually modifying (**a**) *m* and (**b**) *n* on the prediction curves of the conventional JC model.

When only *m* was reduced, the predictions at the reference temperature remained unchanged, as the thermal softening term in Equation (2) remains unity (equal to 1) under such conditions. However, the predictions at elevated temperatures shifted closer to the experimental values (as seen by comparing Figure 6a with Figure 4a). Adjusting the *m* value effectively modulates the vertical spacing between prediction curves at different temperatures but does not alter the slopes of these curves (Figure 7a) (Slope 1 = Slope 2). Conversely, when only *n* was reduced, the prediction curves exhibited a clockwise rotation centered around the point of maximum strain at the reference temperature (Figure 7b). This modification led to a decrease in the slopes of the flow stress curves (Slope 1 > Slope 3). Furthermore, adjusting *n* also influenced the distances between the prediction curves across various deformation temperatures. Consequently, the predictive accuracy of the conventional JC model can be significantly enhanced by appropriately dynamically tuning the values of *m* and *n* based on specific deformation conditions.

**Figure 8:**
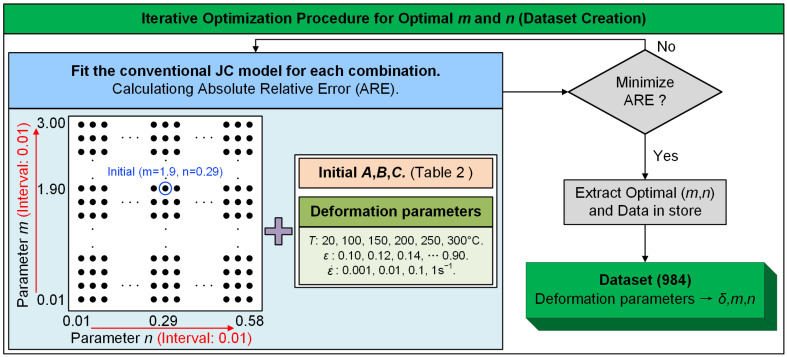
Schematic illustration of the iterative optimization procedure of JC model parameters (*m* and *n*) and dataset construction.

To verify the feasibility of precisely modifying the parameters of the conventional JC model, an iterative optimization procedure was first employed to calibrate *m* and *n*. As illustrated in Figure 8, the parameter search domain was bounded by a lower limit of 0.01 and an upper limit of twice the initial values. Specifically, for each unique combination of temperature, strain, and strain rate, *m* was varied from 0.01 to 3, and *n* from 0.01 to 0.58, both in increments of 0.01. Each deformation condition and all corresponding (*m*, *n*) combinations were substituted into the conventional JC model to calculate the ARE(|*δ*|). The parameter pair yielding the minimum |*δ*| was identified as the optimal (*m*, *n*) solution for that specific deformation state. Given that there are 984 total combinations of deformation conditions, a comprehensive dataset comprising 984 sets of deformation parameters, optimal *m* and *n* values and their associated *δ* was established. Through this iterative optimization procedure, the maximum absolute relative error (ARE) of the predicted flow stress dropped to the order of 10^−6^, representing a substantial improvement in predictive accuracy over the conventional model.

**Figure 9:**
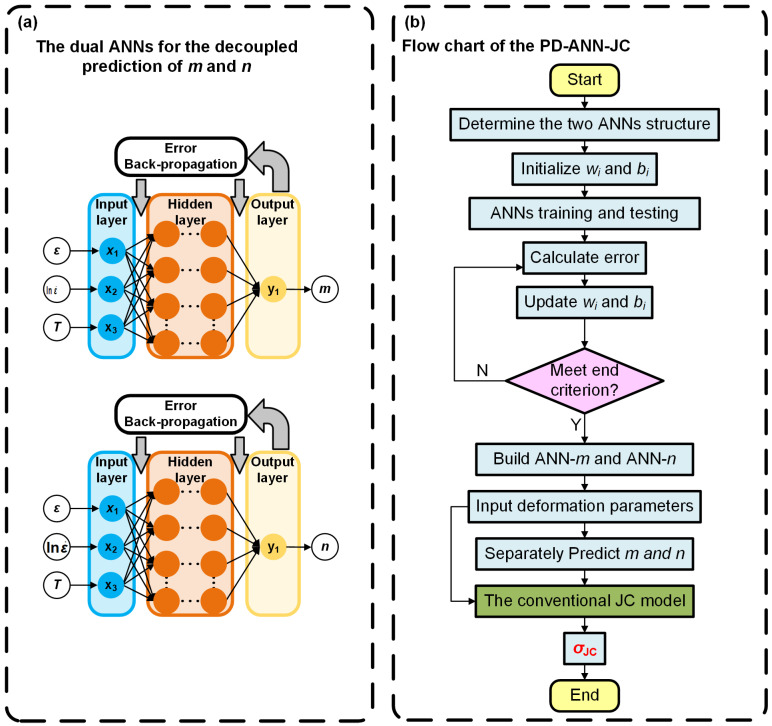
(**a**) Architecture of the three-input, single-output Parallel-Decoupled ANN (ANN-*m* and ANN-*n*) for independent prediction of *m* and *n*, and (**b**) schematic of the coupled PD-ANN-JC framework for flow stress evaluation.

**Figure 10:**
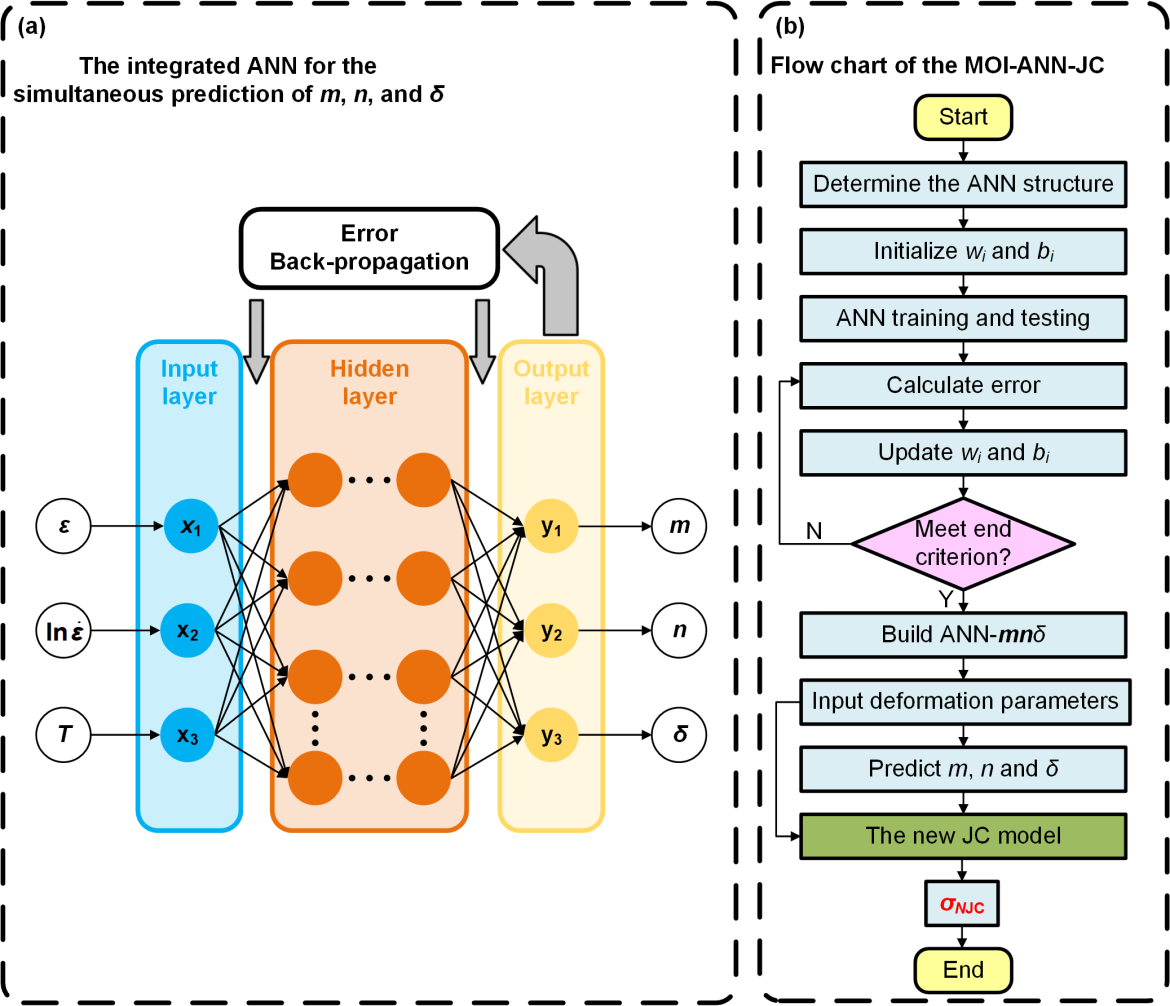
(**a**) Architecture of the three-input, three-output Multi-Objective Integrated ANN (ANN-*mnδ*), and (**b**) flowchart of the MOI-ANN-JC framework based on the modified JC model.

Furthermore, utilizing the established dataset, two types of artificial neural network (ANN) surrogate models were developed to rapidly predict the optimal combinations of *m* and *n*. The first is a three-input, single-output Parallel-Decoupled ANN (PD-ANN; Figure 9a), which independently predicts *m* and *n* based on the deformation parameters (ANN-*m* and ANN-*n*). The second is a three-input, three-output Multi-Objective Integrated ANN (MOI-ANN; Figure 10a), capable of simultaneously predicting both the physical parameters and the relative error (*δ*) of the JC model (ANN-*mnδ*). Based on the PD-ANN surrogate model, a PD-ANN-JC framework was constructed (Figure 9b), wherein the independently derived *m* and *n* values are incorporated into the conventional JC model for flow stress prediction.

Because the MOI-ANN surrogate model can simultaneously output *δ*, a modified JC model (Equation (7)) was formulated to further enhance predictive precision.

(7) σNJC=11−δσJC=11−δ(188+300εn)1−0.0082lnε˙ε˙r1−T−TrTm−Trm

Grounded in this model, the MOI-ANN-JC framework was proposed (Figure 10b), where the neural network takes deformation parameters as inputs to simultaneously generate *m*, *n* and *δ*. Then, these outputs are subsequently substituted into Equation (7) to calculate flow stress.

To evaluate the prediction accuracy and generalization capability of the established frameworks, a rigorous partitioning of the training, validation, and testing sets was conducted. Specifically, data points from the established dataset corresponding to a strain range of 0.1 to 0.7 were designated as the training set to train the surrogate models. Data points corresponding to a strain range of 0.7 to 0.9 were assigned as the validation set to verify the generalization capability of the ML-JC frameworks. From the Gleeble compression test data, deformation parameters and corresponding stress values within the strain range of 0.11 to 0.69 (at intervals of 0.02) were selected as the testing set. This rigorous partitioning ensures that the training, validation, and testing sets are completely independent, thereby completely preventing data leakage.

Prior to training the ANNs, it is essential to normalize both the input and output values to the range of [−1, 1] to enhance training efficiency and convergence stability. The normalization was performed according to the following equation:

(8) $$X_{n}=-1+\frac{2\left(X-X_{\min}\right)}{X_{\max}-X_{\min}}$$

where *X* represents the raw experimental data, while *X_n_*, *X*_max_, and *X*_min_ denote the normalized value, the maximum value, and the minimum value of the original dataset, respectively. Furthermore, by rearranging the normalization expression, the corresponding inverse-normalization equation can be derived as follows. This allows the dimensionless network outputs to be mapped back into their physical dimensions for the actual determination of *m*, *n*, and *δ*.

(9) $$X=X_{\min}+0.5\left(X_{n}+1\right)\left(X_{\max}-X_{\min}\right)$$

The development and training of the ANNs were implemented using Python (Version 3.11.14). To introduce nonlinearity and mitigate the vanishing gradient problem, the Rectified Linear Unit (ReLU) was employed as the activation function for both the transitions from the input to the hidden layers and among the hidden layers themselves. Conversely, a linear transfer function (without an activation function) was utilized from the final hidden layer to the output layer to ensure a continuous range of predicted values. The weight parameters are critical determinants of each neuron’s output, directly governing the convergence speed and predictive accuracy of both the PD-ANN and MOI-ANN models. The output of an individual neuron was calculated according to Equation (10) as follows:

(10) $$y_{o}=g(\sum_{i=1}^{L}w_{i}x_{i}+b_{i})$$

where *L* is the number of input nodes, *x_i_* is the input vector, *w_i_* is the weight vector, *b_i_* is the bias, *g* is the activation function and *y*_o_ is the output. At the onset of the training process, the weight (*w_i_*) and bias (*b_i_*) parameters were randomly initialized. The error signals, representing the discrepancies between the experimental results and the network predictions, were then back-propagated to iteratively update the weights and biases. To determine the optimal architecture for both the ANN models, various configurations of hidden layers and neuron counts were systematically evaluated. The model performance was assessed based on the Mean Absolute Error (MAE), which quantifies the deviation between the predicted values and the experimental data. The MAE is defined by the following equation:

(11) $$\mathrm{MAE}=\frac{1}{M}\sum_{i=1}^{M}\left|y_{i}-\bar{y_{i}}\right|$$

where *M* denotes the total number of samples, *y_i_* represents the experimental value, and $\bar{y_{i}}$ represents the corresponding prediction from the ANNs. To identify this optimal configuration, the number of hidden layers was varied from 1 to 3, while the specific number of neurons within each hidden layer was determined according to the following empirical Equation:

(12) $$S_{2}=\sqrt{S_{1}+S_{3}}+a$$

where *S*_2_ denotes the number of neurons in the hidden layer, *S*_1_ and *S*_3_ represent the number of neurons in the input and output layers, respectively, and *a* is an adjustment constant ranging from 1 to 10.

**Figure 11:**
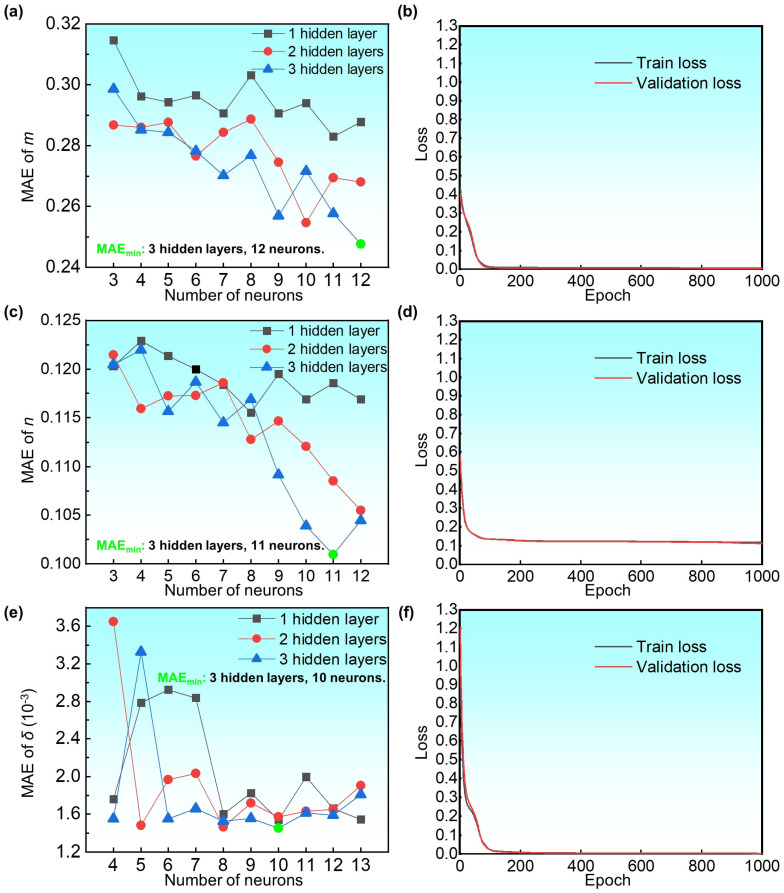
Determination of the optimal ANNs’ structures: (**a**,**b**) ANN-*m*, (**c**,**d**) ANN-*n*, (**e**,**f**) ANN-*mnδ*.

The ANNs were optimized by varying the number of hidden layer neurons from 3 to 12 for the single-output networks (ANN-*m* and ANN-*n*), and from 4 to 13 for the multi-output network (ANN-*mnδ*). By evaluating the Mean Absolute Error (MAE) variations shown in Figure 11a,c,e, the optimal network structures and their corresponding minimal MAE values were determined as follows: 3 × 12 × 12 × 12 × 1 for ANN-*m* (MAE = 0.2476), 3 × 11 × 11 × 11 × 1 for ANN-*n* (MAE = 0.1006), and 3 × 10 × 10 × 10 × 3 for ANN-*mnδ* (*δ* MAE = 1.4247 × 10^−4^). Furthermore, the loss curves (Figure 11b,d,f) confirm that no overfitting occurred in any of the models, establishing 200 epochs as the optimal training length for all three networks.

**Figure 12:**
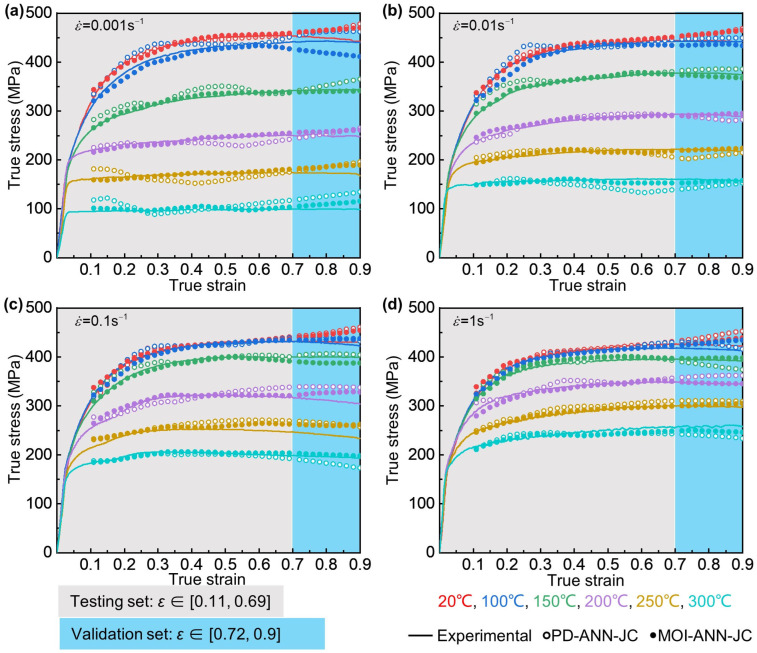
Comparisons between the experimental flow stress curves and the predictions from the PD-ANN-JC and MOI-ANN-JC frameworks under diverse strain rates: (**a**) 0.001 s^−1^, (**b**) 0.01 s^−1^, (**c**) 0.1 s^−1^, and (**d**) 1 s^−1^.

**Figure 13:**
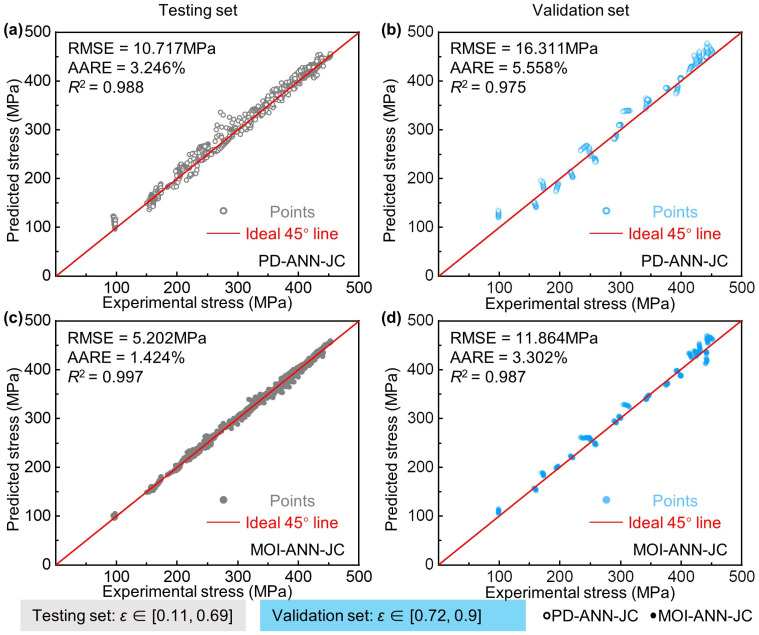
Correlation between the experimental and predicted flow stress for the PD-ANN-JC and MOI-ANN-JC frameworks: (**a**,**c**) testing set and (**b**,**d**) validation set.

Compared to the evaluation of the conventional JC model shown in Figure 5, both the PD-ANN-JC and MOI-ANN-JC frameworks exhibit significantly enhanced predictive accuracy for flow stress across all investigated strain rates (0.001, 0.01, 0.1, and 1 s^−1^) (Figure 12). As summarized in Figure 13a,c, the MOI-ANN-JC framework achieved an RMSE of 5.202 MPa, an AARE of 1.424%, and an *R*^2^ of 0.997 in the testing set. These statistical metrics outperform those of the PD-ANN-JC model, which yielded an RMSE of 10.717 MPa, an AARE of 3.246%, and an *R*^2^ of 0.988 in the same set. Furthermore, the MOI-ANN-JC framework consistently maintains its superior predictive fidelity in the validation set, showing higher *R*^2^ and lower RMSE and AARE values than the PD-ANN-JC approach. Consequently, compared with the PD-ANN-JC framework, the MOI-ANN-JC framework exhibits superior predictive fidelity and enhanced generalizability.

### 3.2. Application in Characterization of Warm Deformation Workability

**Figure 14:**
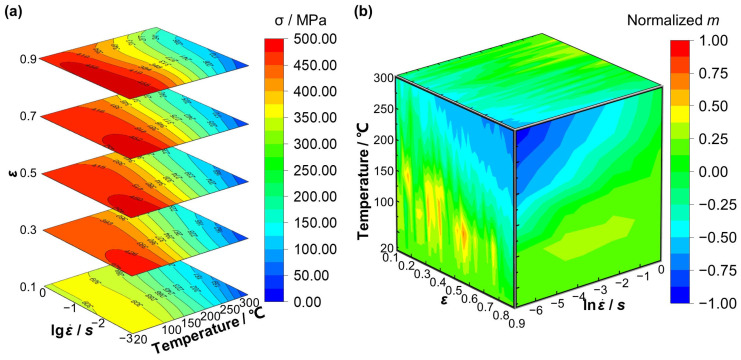
Characterization of the 5A06 aluminum alloy using the MOI-ANN-JC framework: (**a**) predicted flow stress distribution and (**b**) dynamic evolution of the thermal softening exponent (*m*) under various deformation conditions.

By employing the proposed MOI-ANN-JC framework, not only can the warm deformation behavior of the 5A06 aluminum alloy be accurately characterized (Figure 14a), but the evolution of the thermal softening exponent (*m*) under varying deformation conditions can also be dynamically tracked (Figure 14b). To further investigate the intrinsic correlation between the warm deformation behavior and the underlying material properties, the temperature-dependent term in Equation (4) warrants deeper exploration. The distribution of *m* reveals that its minimum values occur under conditions of high strain, low strain rate, and elevated temperature (Figure 14b). Based on Equation (4), at a constant temperature, a smaller *m* value corresponds to a lower flow stress, suggesting that a reduced *m* value may signify a more pronounced thermal softening effect within the material. However, this hypothesis requires further experimental validation.

**Figure 15:**
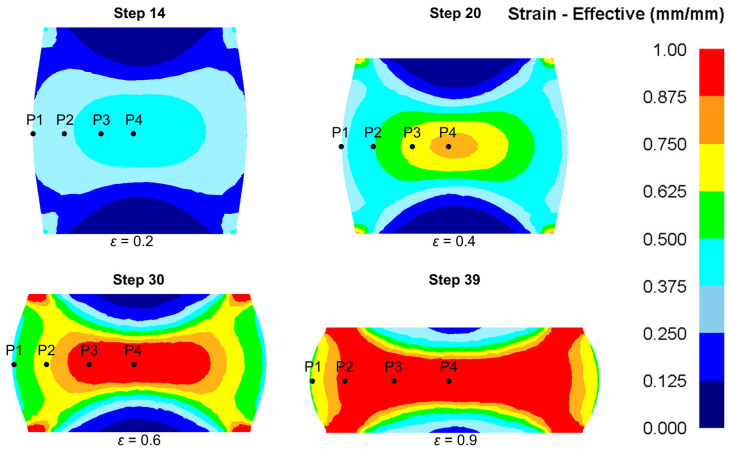
Finite element analysis (FEA) results illustrating the continuous increase in effective strain at specific radial locations (P1–P4) on the longitudinal section of the compressed specimen.

To elucidate the intrinsic correlation between the thermal softening exponent (*m*) and the actual thermal softening behavior of the material, finite element analysis (FEA) simulations and Vickers hardness tests were conducted on the compressed specimens. Initially, the developed MOI-ANN-JC framework was embedded into the DEFORM (v11.0) FEA software via secondary development. This integration facilitated the direct visualization of the predicted *m* field distribution during the post-processing stage. Subsequently, building upon the Gleeble compression testing depicted in Figure 1, supplementary compression tests were executed at 150 °C to achieve true strains (along the compression axis, hereafter simply referred to as true strain) of 0.2, 0.4, and 0.6. Following this, corresponding FEA models of the Gleeble compression tests were constructed. The cylindrical specimen model was symmetrically bisected to expose its internal longitudinal plane. On this exposed surface, point P4 was designated at the exact geometric center. Points P3, P2, and P1 were then assigned at equidistant radial intervals from the center (P4) to the outer surface at the same horizontal elevation. The spatial trajectories of these four tracking points were continuously monitored throughout the simulation. Ultimately, the evolution of Vickers hardness at these four specific locations with varying strain levels was cross-analyzed against the predicted *m* distributions. As the compression deformation progressed, the effective strain in the vicinity of all four points exhibited a continuous increase (Figure 15). Theoretically, the Vickers hardness at these four locations would be expected to increase monotonically due to work hardening.

**Figure 16:**
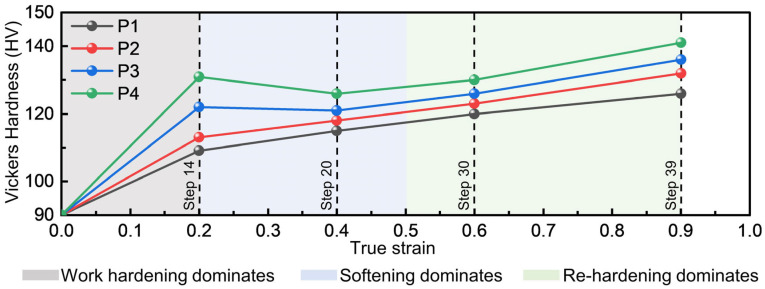
Variation in Vickers hardness at the four designated tracking points (P1–P4) under different true strains during warm compression.

However, as depicted in Figure 16, the evolution of Vickers hardness at the four designated points does not exhibit a simple linear correlation with the increasing effective strain. During the initial stage of compression (true strain ranging from 0 to 0.2), the Vickers hardness at all four locations increases monotonically with the effective strain, indicating a pronounced work hardening effect. As the true strain reaches 0.4, the growth rate (indicated by the slope of the curve) of Vickers hardness at points P1 and P2 decelerates compared to the initial deformation stage. Conversely, the Vickers hardness at points P3 and P4 undergoes a noticeable decline. This dynamic suggests that work hardening remains the dominant mechanism at P1 and P2, whereas thermal softening takes precedence at P3 and P4 during this intermediate stage. When the true strain exceeds 0.4, the Vickers hardness at all four points resumes a linear growth trajectory with respect to the increasing strain. Nevertheless, the rate of this increase (curve slope) is visibly lower than that observed during the initial compression stage. This phenomenon elucidates that both work hardening and thermal softening mechanisms operate concurrently during this advanced phase, with work hardening once again reasserting its overall dominance.

**Figure 17:**
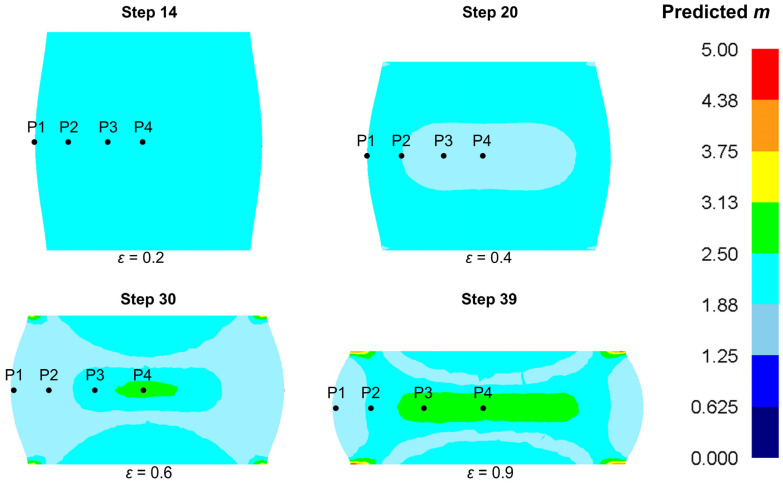
Spatial evolution of the predicted *m* field within the compressed specimen at varying true strains.

The spatial distribution of the predicted *m* field within the compressed specimen is illustrated in Figure 17. During the initial stage of compression, the *m* field exhibits a relatively uniform distribution throughout the material. However, as the true strain reaches 0.4, a distinct localized zone characterized by low *m* values emerges at the core of the specimen. Notably, tracking points P3 and P4 are situated exactly within this low-*m* region, which perfectly corroborates the significant drop observed in their respective Vickers hardness values. Upon further deformation to a true strain of 0.6, this low-*m* zone migrates outward from the specimen’s core toward the surface regions, accompanied by an increase in the *m* value at the core. Correspondingly, the growth rate of Vickers hardness at points P1 and P2 attenuates, whereas the hardness at points P3 and P4 resumes an upward trajectory. Consequently, it can be conclusively deduced that the spatial evolution of the *m* field accurately maps the dynamic localized softening regions within the deformed specimen.

**Figure 18:**
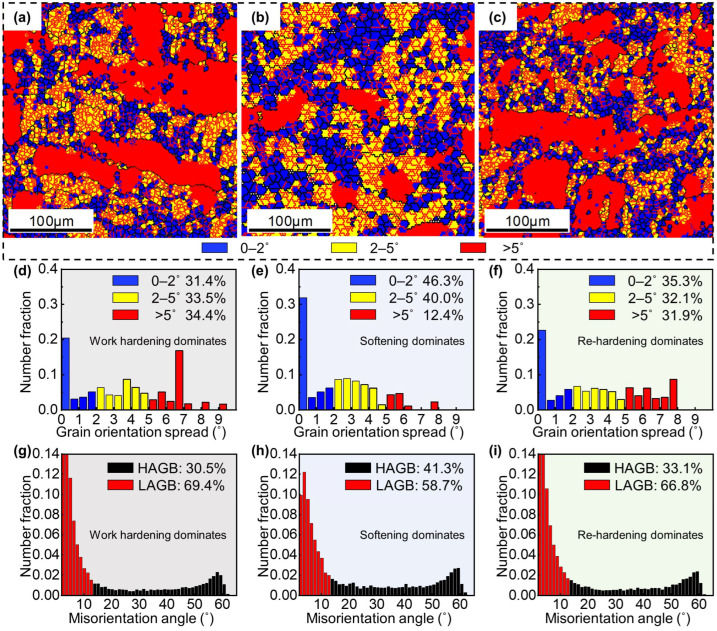
EBSD analysis results at the specimen core (point P4). Grain Orientation Spread (GOS) maps at true strains of (**a**) 0.2, (**b**) 0.4 and (**c**) 0.9; GOS distributions at true strains of (**d**) 0.2, (**e**) 0.4 and (**f**) 0.9; Misorientation Angle (MA) distributions at true strains of (**g**) 0.2, (**h**) 0.4 and (**i**) 0.9.

To further elucidate the underlying softening mechanisms operating during the compression process, detailed microstructural characterizations were conducted. Specifically, specimens deformed to true strains of 0.2, 0.4, and 0.9 were sectioned, and samples were extracted from their core regions (corresponding to the P4 location). EBSD analysis was subsequently performed on these samples to quantitatively evaluate the Grain Orientation Spread (GOS) and the Misorientation Angle (MA) distributions, as presented in Figure 18. The microstructural evolution at the specimen core (point P4) perfectly elucidates the macroscopic competition between work hardening and thermal softening. At an initial true strain of 0.2 (Figure 18a,d,g), the microstructure is heavily dominated by Low Angle Grain Boundaries (LAGBs), which account for 69.4% of the total boundaries, accompanied by a relatively even distribution of misorientation angles (MAs). This high fraction of LAGBs signifies rapid dislocation multiplication and entanglement, directly governing the pronounced work hardening and the corresponding increase in Vickers hardness observed during the early deformation stage. Intriguingly, as the true strain reaches 0.4 (Figure 18b,e,h), a significant microstructural transformation occurs. The fraction of HAGBs increases noticeably to 41.3%. More notably, the MA distribution exhibits a sharp surge in the 0~2° range (46.3%) and a drastic reduction in the >5° range (12.4%). This distinct shift suggests extensive dislocation annihilation and subgrain formation, indicating the occurrence of intense dynamic recovery (DRV) and the onset of dynamic recrystallization (DRX) at the specimen core. This microstructural softening mechanism perfectly corroborates the previously observed decline in Vickers hardness at P4 and accurately aligns with the low *m* region predicted by the MOI-ANN-JC model. Upon further severe deformation to a true strain of 0.9 (Figure 18c,f,i), the microstructural trend reverses. The LAGB fraction surges back to 66.8%, and the MA fraction > 5° recovers to 31.9%. This indicates that sustained plastic deformation induces the generation of new dislocations within the previously recovered or recrystallized grains, leading to renewed sub-grain boundary formation and increased dislocation density. Consequently, work hardening reasserts its overall dominance over thermal softening, which is highly consistent with the resumed growth trajectory of Vickers hardness at advanced strain levels.

**Figure 19:**
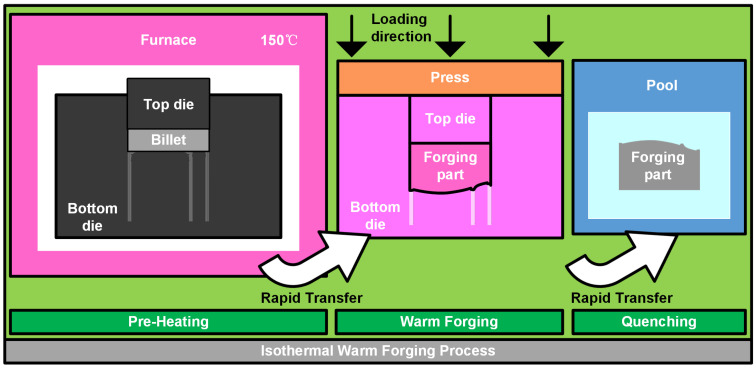
Schematic diagram of the isothermal warm forging process for the 5A06 aluminum alloy component.

Finally, to further validate the applicability of the MOI-ANN-JC framework in actual forging, an isothermal warm forging experiment for a thin-walled, dual-cavity component made of the 5A06 aluminum alloy was designed. Concurrently, a corresponding finite element (FE) simulation model was established for comparative analysis. The schematic representation of the isothermal forging procedure is depicted in Figure 19. Initially, the billet and the dies were assembled and placed into a heat treatment furnace and heated at 150 °C for 8 h together to ensure a uniform temperature distribution across both the dies and the billet. Subsequently, the pre-assembled die setup was transferred to a press for the forging operation. Given that both the dies and the billet were preheated, the heat dissipation from the material during the forging process was considered negligible. Upon completion of the forging, the component was immediately subjected to water quenching. Based on the actual dimensions of the dies and billet, the FE simulation models were constructed, wherein the initial temperatures of both components were set to 150 °C, and the downward velocity of the upper die was maintained at 2 mm/s.

**Figure 20:**
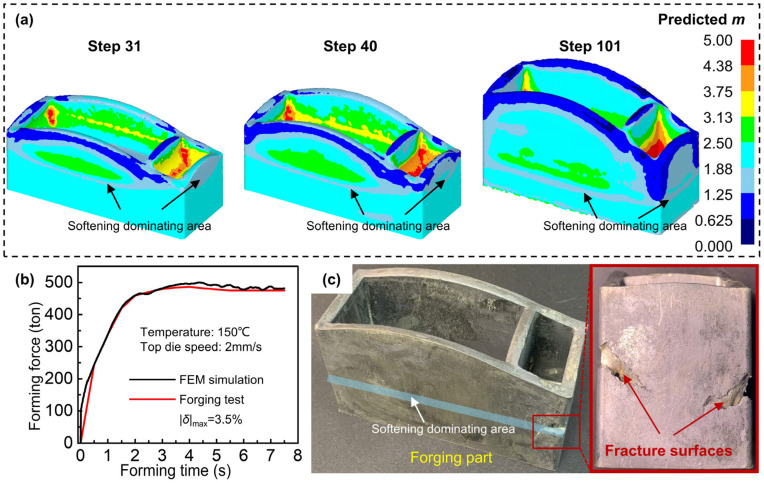
Comparison between the FE simulation and experimental results for the isothermal warm forging of the dual-cavity component: (**a**) distribution of the predicted *m* field and (**c**) the corresponding macroscopic fracture, and (**b**) evolution of the forming force.

The finite element (FE) simulation and the corresponding experimental results for the isothermal warm forging of the dual-cavity component are presented in Figure 20. Specifically, Figure 20a illustrates the distribution of the *m* field on the billet during the deformation process. In the initial stage of forging, a distinct region characterized by low *m* values (indicated in grey) emerges on the outer surface of the forging, which persists throughout the entire forging process. As elucidated in the preceding sections, the material within this specific zone experiences severe softening, predominantly driven by dynamic recrystallization (DRX) and dynamic recovery (DRV). Strikingly, on the actual forged component, a macroscopic fracture surface appeared precisely at the location corresponding to this low-*m* zone (Figure 20c). The observation indicates that localized softening during the warm forging process severely compromises the structural integrity of the forging and must be strictly avoided through process optimization. Furthermore, the forming force evolution curve obtained from the physical forging experiment aligns remarkably well with the FE simulation results, exhibiting a maximum error of merely 3.5% (Figure 20b). This high degree of consistency robustly demonstrates that the established MOI-ANN-JC framework can accurately predict the forming force and capture the complex deformation behavior of the 5A06 aluminum alloy during actual warm forging operations.

## 4. Conclusions

In this study, machine learning-assisted Johnson–Cook (ML-JC) frameworks based on artificial neural network (ANN) surrogate models were developed and systematically validated to characterize the warm deformation behavior and workability of the 5A06 aluminum alloy. The principal conclusions are drawn as follows:

(1) Compared with the conventional JC model, both developed ML-JC frameworks (MOI-ANN-JC and PD-ANN-JC) achieve significantly higher stress prediction accuracy and superior generalization capability. Specifically, on the testing set, the MOI-ANN-JC framework yields an AARE of 1.424% and an *R*^2^ of 0.997, whereas the PD-ANN-JC framework yields an AARE of 3.246% and an *R*^2^ of 0.988. On the validation set, the AARE and *R*^2^ are 3.302% and 0.987 for MOI-ANN-JC, and 5.558% and 0.975 for PD-ANN-JC, respectively.

(2) By adopting the ANN-*mnδ* surrogate model, the MOI-ANN-JC framework simultaneously predicts *m*, *n*, and *δ* directly from the deformation parameters, thereby establishing an intrinsic correlation between *m* and *n*. This mutual coupling aligns with the physical reality wherein strain hardening and thermal softening are inherently linked during deformation. Consequently, its prediction performance outperforms that of the PD-ANN-JC framework, which predicts *m* and *n* independently.

(3) The MOI-ANN-JC framework was integrated into finite element (FE) simulation software to dynamically track and visualize the thermal softening exponent *m* during warm deformation. Combined with FE simulations, Vickers hardness testing and EBSD observations, this study successfully establishes a qualitative spatial correspondence between low-*m* regions and macroscopic defects. The validity of this correspondence was further verified through the warm forging of a thin-walled dual-cavity component. Crucially, this approach for evaluating deformation stability bridges the gap caused by the inapplicability of conventional processing maps within this temperature regime.

(4) The research workflow established in this study—which encompasses dataset creation for modified parameters, surrogate model training, construction of the surrogate-driven JC framework for flow stress prediction, rigorous validation of accuracy and generalization, and practical application in forming processes—possesses broad applicability. Nevertheless, the quantitative relationship between the thermal softening exponent *m* and material instability during deformation requires systematic investigation in future work to establish a comprehensive stability evaluation framework for warm deformation.

## Figures and Tables

**Table 1:** Material composition details of the 5A06–H112 aluminum alloy (Wt.%).

5A06	Mg	Si	Fe	Cu	Mn	Zn	Ti	Be	Al
Wt.%	5.9	0.06	0.15	0.01	0.61	0.01	0.05	0.0008	Remainder

**Table 2:** Identified parameters for the conventional Johnson–Cook (JC) model.

${\dot{\varepsilon }}_{r}$	*T_m_*	*T_r_*	*A*	*B*	*n*	*C*	*m*
0.001 s^−1^	550 °C	20 °C	188	300	0.29	−0.0082	1.9

**Table 3:** Max absolute relative errors of the conventional JC model under different deformation conditions.

	$\dot{\varepsilon }$ = 0.001 s^−1^	$\dot{\varepsilon }$ = 0.01 s^−1^	$\dot{\varepsilon }$ = 0.1 s^−1^	$\dot{\varepsilon }$ = 1 s^−1^
**|*δ*|_max_**	70.62%	52.39%	41.45%	18.59%

## Data Availability

The original contributions presented in this study are included in the article. Further inquiries can be directed to the corresponding authors.
